# Increasing reach of cancer care: provider perspectives on the value and use of teleoncology

**DOI:** 10.1186/s12913-026-14467-5

**Published:** 2026-04-11

**Authors:** Leah L. Zullig, Abigail Shapiro, Madeleine R. Eldridge, Christa Tumminello, Ivonne Guzman, Scott E. Sherman, Danil Makarov, Daniel Becker, Vida Passero, Navid Dardashti, Michael J. Kelley, Karen Steinhauser

**Affiliations:** 1 Center of Innovation to Accelerate Discovery and Practice Transformation, Durham VA Healthcare System, 411 West Chapel Hill Street, Suite 600, Durham, NC 27701 USA; 2 Department of Population Health Sciences, Duke University School of Medicine, Durham, NC USA; 3 Duke Cancer Institute, Duke University Medical Center, Durham, NC USA; 4 VA New York Harbor Healthcare System, New York, NY USA; 5 Department of Population Health, New York University Grossman School of Medicine, New York, NY USA; 6 University of Pittsburgh School of Medicine, Pittsburgh, PA USA; 7 Veterans Affairs Pittsburgh Healthcare System, Pittsburgh, PA USA; 8 National Oncology Program, Department of Veterans Affairs, Washington, DC USA; 9 Division of Hematology and Oncology, Durham VA Healthcare System, Durham, NC USA

**Keywords:** Veterans health, Telehealth, Care quality, Health services research

## Abstract

**Purpose:**

In-person cancer care within the national Veterans Health Administration (VA) system is challenging for many Veterans due to issues including rurality, transportation, finances, proximity to subspecialists, and other factors. The National TeleOncology Service (NTO) is a clinical service that enables VA oncologists from across the country to provide services via telehealth to Veterans with cancer. We sought to understand NTO providers’ perspectives on the role of the NTO in providing cancer care to Veterans via telehealth.

**Patients and methods:**

We recruited NTO providers through their monthly office hours meeting for two focus groups held via Microsoft Teams in November 2023. We entered data from notes, transcripts, chats, and audio recordings into a matrix for analysis according to question topic and associated Consolidated Framework for Implementation Research domains. We used a rapid qualitative analysis approach.

**Results:**

Providers (*n* = 23) widely agreed that NTO service provides valuable cancer care, particularly for Veterans facing in-person access issues across many phases of cancer treatment and survivorship. NTO services include multidisciplinary providers in patient calls and make it easier to include patient caregivers who may not be able to travel to appointments. Despite initial rapport building being somewhat challenging, providers still reported that NTO overall is of high value, particularly for rural Veterans utilizing VA-provided cancer care.

**Conclusion:**

Remotely delivered cancer care is an important addition to cancer care services. This may be especially important for Veterans living in rural areas or far from needed cancer care services. VA providers offer an important perspective on the role of NTO in cancer care, the barriers they face to delivering that care, and the facilitators that VA can utilize to promote the success of NTO. In an era when Centers for Medicare & Medicaid Services (CMS) shifts away from reimbursing telehealth, the VA has committed to continue such care providing a variety of patient-centered approaches.

## Introduction

Cancer remains the second leading cause of American deaths [1]. Additionally, the U.S. is experiencing rising incidence of cancers, including early-onset cancers or cancers involving younger people [2]. Critical to combating this issue is timely access to cancer treatment, which necessitates access to oncologists. However, there is a shortage of oncologists across the U.S., and that shortage is particularly pronounced in rural areas; two-thirds of rural counties lack an oncologist [3, 4]. The American Society of Clinical Oncology (ASCO) has reported that that workforce shortage leaves about 32 million Americans without an oncologist nearby [3, 4]. This shortage has been a particular problem for care within the Veterans Health Administration (VA), since approximately one-quarter of Veterans live in rural areas [5]. This rise in early onset cancers, coupled with the shortage of oncologists overall and especially in rural areas, has reached a precipice where many people lack access to critical cancer care services close to their homes. One potential solution is to increase cancer care access via telehealth.

In the U.S., there has been a tremendous increase in telehealth use for cancer care. Initially, this increase in telehealth was driven by the COVID-19 pandemic, when telehealth use peaked at 34% of visits in 2020 [6]. Since this spike in telehealth-delivered care, rates have now steadied at approximately 15% [6]. There are several potential reasons for the sustained interest in telehealth care even after the pandemic subsided. Evidence suggests that telehealth may overcome many traditional barriers to in-person care, thereby increasing care access [7, 8]. Telehealth can also improve care quality, communication between patients and their care teams, and health outcomes including symptom management and medication adherence, among others [9–11]. While telehealth care widely has been used for primary care and mental health services, there is increasing support for the use of telehealth in cancer care delivery and related research efforts [6, 12, 13]. Many aspects of cancer care – including supporting healthy lifestyle for cancer prevention and screening, remotely monitoring patients with cancer for symptoms and side effects during cancer treatment, and virtual survivorship care – can be provided remotely [11, 12]. 

The national Veterans Health Administration (VA) is an innovator in telehealth-delivered cancer care. Prior to the COVID-19 pandemic, the VA’s National TeleOncology Service (NTO) was established to provide specialized treatment for cancer to Veterans regardless of their geographic location [14]. The NTO delivers cancer screening, diagnostics, and treatment for medical oncology including genetic screening, rehabilitation, and palliative care. The NTO has established disease-specific teams to support sub-specialized cancer clinics for malignant hematology, thoracic, health and neck, genitourinary, benign hematology, gastro-intestinal, and rare cancers. These disease-specific teams include physicians, an advanced practice provider, clinical pharmacist, and a nurse care coordinator. The NTO was initiated in 2020. In the first three years of its existence, nearly 5,000 Veterans received cancer-related services through NTO. In addition to being a leader in terms of the volume of cancer care provided via telehealth, the VA also provides a unique and important context in which to study telehealth-delivered cancer care for two key reasons. First, the VA determines many aspects of its financial models and is not reliant on Centers for Medicare & Medicaid reimbursement policies. Specifically, the VA continued delivery of telehealth care even after Medicare decreased its reimbursement for such services. Second, because it continued to provide a high volume of post-pandemic telehealth delivered care, the VA is well positioned to assess experiences with that care. To inform programmatic decision making, we sought to explore the perspectives of VA physicians and non-physician staff members regarding contextual factors impacting cancer-related telehealth service implementation and sustainment at multiple levels. Specifically, we sought to understand the physical and sociocultural environments, as well as health care system factors, impacting the success of cancer-related telehealth-delivered care across the national VA health care system.

## Methods

We conducted purposive sampling of physicians and non-physician staff members providing care through the NTO. Prior to initiating qualitative data collection, the lead study investigator (LZ) introduced the physicians and non-physicians to the project and invited them to join a 30-minute focus group during a regularly occurring NTO weekly standing. To be mindful of professional dynamics and supervisory role considerations, we held separate focus groups for physicians and non-physicians. Focus groups included trained facilitators and notetakers (AS, IG, CT, LZ).

Participants were asked to provide verbal consent prior to participation. We conducted, recorded, and auto-transcribed the focus groups using Microsoft Teams. Our research team used a structured note-taking process during the focus group and verified notes using the recordings. We used a team-based rapid qualitative analysis approach [15] to understand provider perspectives on the contextual factors impacting provider and Veteran experiences of cancer telehealth at VA.

Our interview guide was developed by a multidisciplinary research team with qualitative methodology expertise. The Consolidated Framework for Implementation Research (CFIR) guided our consideration of contextual factors that might impact virtually delivered cancer care, and our semi-structured interview guide and analysis plans were based upon select domains and constructs of that framework [16]. The study was approved by the Durham Veterans Affairs Health Care System IRB Committee. All participants provided informed consent. The study was conducted in compliance with the Declaration of Helsinki. We do not report identifying images or other personal or clinical details of participants and have included a statement and consent for publication is not applicable.

### Analysis

We used rapid qualitative analysis procedures to collect and analyze qualitative data [17–19]. Our analytic approach included utilization of matrix frameworks [20]. Matrices were derived from the theoretical constructs and the aligned focus group questions to reduce, summarize, and compare data within and across sampled groups (e.g., physician and non-physician participants). We abstracted the data and summarized it into memos by domain. The research team met to review matrices and consider programmatic relevance, and subsequently reviewed transcripts to verify summaries and identify quotations. We also considered professional role, physician type, and length of time with NTO of respondents when applicable. We managed and analyzed data in Microsoft Excel and NVivo (version 12, 2017).

## Results

We conducted two focus groups of participants in the NTO Hub Office Hours Call in November 2023. We invited all members of the NTO team, which created differential focus group sizes. One focus group was comprised of physicians (*n* = 4). The other focus group was comprised of non-physicians (*n* = 19) including clinicians with credentials in nursing, mid-level providers, pharmacists, and non-clinician others. The physician focus group lasted 24 min, the non-physician group lasted 31 min.

Below we present the qualitatively derived concepts and exemplar quotes (Table 1); quotes are organized by those that represent barriers or facilitator to uptake, those impacting Veterans or Providers, and noting the associated CFIR domains [16]. These included constructs within the following domains:

**Table 1:** Representative quotes from Veterans and clinicians illustrating the values, barriers, and facilitators of National TeleOncology Service (NTO), mapped to the Consolidated Framework for Implementation Research (CFIR)

Concept(s)	Who is Affected	Quote	CFIR Domain(s)
Value	Veteran	“But I do think that it allows for patients who may have struggles like and used all their resources to come in for their radiation treatment. If we’re allowed to give them telehealth encounters for follow up, I think it helps them so that they doesn’t tax their network who helps to bring them in for appointments.”	• Innovation Domain: *Innovation Relative Advantage*
Value	Veteran & Clinician	“I think one positive of the telemedicine I found in my experience is that not only am I able to focus a little more on just that one patient that is in front of me, but I’m able to give them the full amount of time allotted for their appointment I don’t feel rushed as much as in clinic when everything is on fire around you and you’re having to do many different things.”	
Barrier	Veteran & Clinician	“Not really a downside, but I think it’s more challenging to establish a relationship with the patient, especially as a pharmacist when you’re kind of coming on later, maybe a little bit later after they’ve seen the doctor and know what their plans gonna be. I feel like you do have to work a little harder to kind of establish that trust with them and, it does take a little bit of extra work starting out, but, after that it’s great.”	
Barrier & Value	Veteran & Clinician	“So, I think from me, I missed that maybe that a little bit of the emotional connection. You know when you’re sitting in front of your patient and can talk with them, you know about everything they’re going through. It’s a little more - It’s a little bit harder being on the video where you could be right there in front of them, so that’s one of the things that I find a little bit harder.”	
Facilitator	Veteran	“I think the patients are most successful if they’re doing like a CVT [Clinical Video Telehealth] and they go to a clinic and they do that and as they were referencing, if there’s somebody there to help them with it, I think the VVC [VA Video Connect] is the one where the patient can be home and that’s what I’ve been referencing in terms of not being able to have that access etcetera.”	• Innovation Domain: Innovation Complexity
Appropriateness & Facilitator	Veteran	“Most people are pretty comfortable, but you’ll have a few that struggle to get on the video. So we’ll call them and you can still do a group phone call that we’ve done previously.”	• Innovation Domain: *Innovation Complexity* • Inner Setting Domain: *Information Technology Infrastructure*
Facilitator	Veteran & Clinician	“What seems to make it work best is if there’s an adequate number of nursing staff. Both provide the patient care. Um, the treatments and the infusion room, if that’s what’s being done. Uh, but like (NAME) said, to help facilitate the actual telehealth visits. I work at probably 9 plus different sites so there’s a lot of variability in in the staffing level and how well they’re able to support the visits. But the ones that work best are the ones that have someone who can be spend more time operating the Tele presenting function and being with the patient.”	• Implementation Process: *Reflecting & Evaluating – Implementation* • Implementation Process: *Assessing Context* • Inner Setting: *Available Resources (Funding*,* Space*,* Materials & Equipment)*
Facilitator & Barrier	Veteran	“I feel like it might have been the (cancer charity). I don’t remember, but donated a large fund of some of money for the social workers to use to give out to patients for gas money. So that was very helpful for the few patients that either they didn’t have gas money or whatever. So our gas cards or taxi vouchers, but more social workers to help us, like find those resources so that we can have them for NTO and you know yeah, there’s not an easy way to get them the iPads to do the visits that I found (…)”	• Inner Setting: *Available Resources (Funding*,* Space*,* Materials & Equipment)* • Inner Setting: *Access to Knowledge & Information* • Inner Setting: *Available Resources (Funding*,* Space*,* Materials & Equipment)* • Inner Setting: *Access to Knowledge & Information*
Barrier & Value	Clinician	“And we need a high level of technical support to make it happen. (…) just for example, been working in a clinic where the power went out. And if you’re in a room with the patient, you can always turn on a flashlight and continue what you’re doing. And so all you need is a flashlight. But for TeleOncology to work, you need a high level of technical support, both for the (…) provider on one end with their devices and connections, and then the site on the other end. So you need people to be able to operate everything you need to be able to troubleshoot in real time and have backup methods to continue the visit should one of those things break down.”	
Barrier	Clinician	“Standardization across the sites is something that I think is a barrier that we all face. We’re all in multiple VA’s at the same time and every system looks a little bit different. So, technology platforms that allow for you to maybe see your view alerts across all of the sites and not have to log in. So things that have, like, a read and write capability to help streamline some of that beefing up some of our ancillary support services like [Name] mentioned, so like social workers and dietitians and some of those things that I think were we’re growing into. I very much echo the ability to get education or information more readily to patients when it’s a CVT [Clinical Video Telehealth] visit and they’re in the clinic, we do have a way because you can drop that into the chat and a nurse can print it out. But when they are at home, there is that additional barrier and then you’re always wondering like, what are the rules? Can I email them? Can I text them? How do I get this information to them so more streamlined system there.”	
Facilitator & Barrier	Clinician	“So now maybe a just maybe pie in the sky, but [I would want] a national integrated health record. There are many, many instances where, you know, day before visit or even 5 minutes before a visit, we’re still scrambling and asking somebody to call some office and fax over a record that’s (…) really relevant to what we need to do that day. So, whatever could be done to make that access of records across multiple different sites and systems and electronic health records would be would really help scale this up to, you know, multiple states and regions.”

- Innovation Domain, which relates to the components of NTO implementation and care provided by the NTO being implemented.
- Inner Setting Domain, which describes the NTO implementation setting (i.e., VA systems and facilities).

Implementation Process Domain, which includes the NTO implementation activities and strategies.

### Perspectives on when telehealth-delivered cancer care is appropriate (Appropriateness)

Participants identified multiple factors impacting whether a given Veteran was appropriate for using NTO. First, participants described the importance of whether the veteran has been treated for a condition covered by a sub-specialty of a treating physician within the NTO. For example, if a Veteran needs care for breast cancer, there are several physicians providing breast cancer care through NTO, so service need and availability may be well aligned, making a Veteran appropriate for NTO.

Both physicians and non-physicians named other Veteran clinical characteristics that influenced designation as appropriate for NTO services including: requiring continuity of care, receiving concurrent treatment with chemoradiation, and being strong candidates for clinical trials not available locally. Conversely, veterans may be perceived as not NTO appropriate if they were: receiving care by a local oncologist, whether VA or community-based; receiving care for multiple concurrent cancer types; or whose regimen requires frequent transportation (e.g., active chemotherapy) (Table 1). Of note, while the majority of Veterans receiving care in NTO have a cancer diagnosis, there are some Veterans with benign hematology needs that may be candidates for NTO care. In benign hematology, many NTO consults are downgraded to local e-consults to save the Veteran travel time into the clinic for NTO interactions.

Veteran’s appropriateness for NTO and the subsequent coordination of their care (e.g., in person VA cancer care, telehealth care, community-based care) was determined by on-site local personal prior to referral for NTO telehealth-delivered cancer care including telechemotherapy. With regard to clinician patient relationship, participants perceived that initiating NTO care earlier or at beginning of care was better for clinician-Veteran relationship building and care options. While earlier was better, physicians in particular noted that it was acceptable to initiate NTO care anytime throughout the care continuum, with the exception of end-of-life care management.

### Perspectives on the value of telehealth-delivered cancer care (Value)

Overall, participants described significant added value of telehealth-delivered cancer care, with significant benefits of the NTO for both Veterans and the clinical care teams. Those value benefits included conservation of veteran resources (e.g. time, money, social network support), ease of incorporating caregivers and loved ones into a clinical encounter, increased comfort and privacy, and the ability to share aspects of a patient’s home environment with their clinicians (Table 1). Both patients and clinicians reported that some Veterans with cancer might especially benefit from the privacy afforded by tele-oncology. For example, Veterans with breast cancer may benefit from an increased perception of safety during their care, as some Veterans may feel intimidated utilizing in-person services for breast care in a male-dominated VA space. For Veterans for whom in-person care is difficult (due to disease stage/symptoms/etc.), participants agreed that virtual care is sufficiently uncomplicated enough to enable continued contact with their provider through VA Video Connect (VVC), along with seeing the Veteran through in-home visits.

For non-physicians, the benefits of telehealth-delivered cancer care included being able to include caregivers, many of whom may be geographically distant from the patient, in a clinical encounter. Physicians also reported that telehealth-delivered care broadly, and NTO-delivered care specifically, facilitated real-time (or concurrent) patient access to other providers for second opinions and supportive services.

While both groups reported many benefits of telehealth-delivered cancer care, they also noted disadvantages. Several participants noted that it was more challenging to build rapport and cultivate trust with Veterans in telehealth-delivered care, especially those whom they had not first had an initial, in-person visit (Table 1). Relatedly, non-physicians reported that providing emotional support was more challenging in a remotely delivered care setting (Table 1). Additionally, physical exams are not possible over video or telephone visits. Logistically, a few non-physicians reported increased distractions and interruptions when working in shared clinical spaces and workrooms.

### Facilitators for NTO

Participants identified logistical, technical, staff, and policy factors that facilitate the work of the NTO. Telehealth-delivered cancer care via video call is most successful when the Veteran can come to a clinic, so that a VA employee (e.g., medical support assistant, telehealth technician, or nurse practitioner) can assist with technical issues (Table 1). Creating a strong, appropriately staffed, and diverse team of medical professionals such as nurses, oncologists, pharmacists, and nurse practitioners is crucial for providing optimal patient care within the NTO (Table 1). Many participants emphasized the need for robust technical support for on-site and remote clinicians, providing real-time troubleshooting capabilities and backup methods to sustain patient consultations seamlessly, particularly in the event of network or equipment failures. Resources needed for scale-up include funds available to individual Veterans to assist with needs (e.g. gas money for Veterans traveling to a local clinic to connect virtually with a provider at a distant VA) and increased availability of social workers to work with Veterans and coordinate resources.

### Barriers for NTO

Physicians and non-physicians described a range of barriers to implementation and scaling up of telehealth-delivered cancer care. While most Veterans were comfortable with video conferencing, a minority encounter difficulty connecting. To accommodate those having connectivity or occasional technical issues, both individual and group phone calls are used as alternatives to video conferencing (Table 1). Modality did not affect the provider’s ability to incorporate additional providers in an unexpected urgent situation. Telehealth-delivered VA cancer care can be accessed by Veterans at home on a personal or VA-provided device, or at VA facilities. While in-clinic and home virtual care are both common modes to connect with services via telehealth, there are others as well. One other mode for connecting with telehealth services are community-based connection options or kiosks, such as a historical partnership with Walmart [21] or at the Veterans of Foreign Wars of the United States and other locations. Participants reported that they rarely see Veterans utilizing Walmart kiosks to connect (Table 1).

Participants also reported barriers to accessibility of the technology needed to scale-up NTO, especially regarding integration of technology across health care systems. Specifically, technology and infrastructure resources needed to scale up telehealth delivered cancer care delivery and the NTO include provider access to records across multiple sites, systems, and electronic health records (EHR) (Table 1). Both physicians and non-physicians wanted EHR data for care provided at any VA facility and/or care provided in the community and purchased by the VA to be integrated and accessible to them. Participants cited reasons such as inability to locate labs and imaging in a timely manner that is needed to make recommendations and frequently scrambling for assistance to get these records from other offices, often moments before a visit.

Participants also noted challenges delivering tele-oncology to Veterans outside their usual geographic area of practice (Table 1). Among telehealth physicians, several reported differences in the experience of providing telehealth-delivered cancer care for Veterans who received care at their “home” site and those that were from geographically distant sites. As a specific example, when NTO physicians were providing telehealth-delivered cancer care, they noted that care was smoother when the NTO physicians and other professionals personally knew the care team at the local site with whom they were placing a consult. When caring for patients at other sites, it was noted to be more challenging for the NTO physicians when they did not share a close professional network with the local site. When treating “local” Veterans, physicians reported that they had more context of personal experiences with referring providers to help them interpret clinical notations in the EHR. One physician noted that they have been with NTO long enough to start to get a feel for some of the referring clinicians at other sites, but that the process to develop a working relationship takes considerable time.

Barriers to scaling telehealth-delivered cancer care included the lack of standardization across sites, due to multiple systems across VA locations, varying levels of ancillary staffing, and lack of a cohesive system to get information to patients at home (Table 1). Participants also noted some Veterans’ lack of technology. (Table 1). While the VA has an iPad provision program [21], clinicians reported that some Veterans may face the barrier of on-site technology pick up. Additionally, some Veterans need extensive help with technology, and supporting resources may be limited (Table 1).

## Discussion

Telehealth delivered health care has become ubiquitous in the U.S. Identifying facilitators and barriers to telehealth delivered oncology care and formulating subsequent solutions is essential to optimal health care delivery. The VA has long been lauded as an equal access health care system. With its National TeleOncology Service, the VA is at the forefront of telehealth delivered cancer care services [14, 22–24]. In our qualitative study, we explored the perspectives of VA NTO physicians and clinical care team members regarding contextual factors impacting cancer-related telehealth service implementation and sustainment at multiple levels. We learned that VA physicians and non-physicians providing telehealth-delivered cancer care feel strongly that the nationally-provided telehealth cancer care is an important and valuable resource for Veterans, especially those in rural areas without an oncologist. Moreover, participants reported that telehealth is a crucial augmentation to existing in-person care. Most Veterans are successful bridging telehealth connections with little or modest support. Participants also reported that they liked providing telehealth-based cancer care because they thought that it met Veterans’ needs. This is consistent with a prior program evaluation which reported that Veterans and their clinical care teams viewed the National TeleOncology Service favorably [25]. Clinicians reported that telehealth visits were best when there had already been an in-person visit for the clinician and Veteran to build rapport.

Compared to in-person care, participants reported distinct barriers for Veterans accessing telehealth including: (1) inaccessibility of a device that would enable them to connect to video-based care (e.g., laptop, iPad, etc.); (2) insufficient comfort with technology to make a telehealth connection. These barriers impacted a small group of Veterans and were viewed as addressable concerns. For example, the physicians mentioned a national program in which a clinician can “prescribe” an iPad for a Veteran needing a device to access telehealth [21]. iPads generally may picked up in person at a VA facility or can be mailed to a Veteran. This approach conflates the barriers traditionally associated with in-person and telehealth delivered care together. For Veterans who lack technology comfort, non-physicians in our study reported success with initiating a group telephone call to trouble-shoot connection issues before resuming video-based telehealth. While not mentioned by participants in our research, there is also a peer support program where trained Veterans can assist other Veterans who are new to using telehealth.

In terms of key facilitators, participants reported that there are numerous workable staffing models, but it is essential to have a dedicated staff effort to support telehealth. Another noted facilitator was the national, integrated electronic health record (EHR). The national EHR supported care coordination across specialties as well as in-person and telehealth-delivered care.

Since some time has passed since our initial data collection, the NTO has made several changes to address barriers and improve service delivery. As an example, participants noted challenges in delivering tele-oncology to Veterans outside of their usual geographic area of practice. NTO has developed relationships with field-based staff through follow-up meetings and has strengthened working relationships between NTO providers and hub staff. Additionally, NTO has addressed VA electronic health record access for all facilities that have an NTO service agreement, which fosters communication. NTO continues to experience delays in receiving information from the community and partners with VA facilities’ community care staff to obtain that information. Another area where we identified a barrier that has been addressed is with a lack of a cohesive system to get information to patients at home. NTO has improved operations is through use of virtual care coordination nurses to reach Veterans at home. NTO is responsive to the needs of Veterans and their care teams and is engaged in continuous growth and improvement strategies.

Our study has three key limitations. First, our focus groups were conducted with a sample of physicians and non-physicians working with the National TeleOncology Service (NTO). It is possible that VA employees who are willing to work with NTO have a more favorable view of telehealth than those who retain an entirely in-person workload. Second, one of the focus groups has a relatively large number of participants (*n* = 19), which may have made it difficult for some participants to voice their ideas. Our trained facilitator attempted to overcome this limitation by inviting participants to also use the chat function. Additionally, we had a relatively small (*n* = 4) group of physician participants. Physicians play an important role in NTO delivery and future studies should seek their input. Furthermore, Veterans were not included as participants in this study. Clinicians often reported their perceptions of the Veteran experience, which may not reflect Veterans’ true experience and is a potential limitation. Finally, as the VA is a large integrated health care system with a substantial track record of providing telehealth-delivered care, our findings may not be transferable to less integrated systems. The VA maintains its own financial model and is not subject to Centers for Medicare & Medicaid Services reimbursement decisions. As a result, if the VA determines that providing telehealth-delivered cancer care is in the best interest of Veterans and the health care system, it can establish its own program design. VA clinicians may practice care across state lines without specific licensure in each state; this enables that VA to expand its reach to Veterans with cancer [26]. The VA also provides opportunities for clinicians to communicate and coordinate care information without being in the same geographic space via the national EHR and the VA’s national virtual tumor board [22]. VA telehealth delivered cancer care is upheld by this continuity and coordination.

Despite these limitations, we assert that learning from the VA as a “living laboratory” of what could be possible for telehealth delivered oncology care makes a substantial contribution. Moreover, as the oncology workforce shortage continues [27, 28] and telehealth use for cancer care reaches a steady state [6], health systems must understand and address barriers to telehealth delivered cancer care.

## Data Availability

No datasets were generated or analysed during the current study.

## Declarations

### Previous presentation

The work was previously presented as a roundtable discussion at the Association of Public Health (APHA) annual meeting in October 2024 in Minneapolis, MN.

### Disclaimer

The contents do not represent the views of the US Department of Veterans Affairs or the US Government.

## Abbreviations

ASCO: American Society of Clinical Oncology
CFIR: Consolidated Framework for Implementation Research
CMS: Centers for Medicare & Medicaid Services
HER: Electronic Health Records
IRB: Institutional Review Board
NTO: National TeleOncology Service
VA: Veterans Health Administration
VVC: VA Video Connect

## References

[1] Bray F, Laversanne M, Weiderpass E, Soerjomataram I. The ever-increasing importance of cancer as a leading cause of premature death worldwide. Cancer. 2021;127(16):3029–30. 34086348 10.1002/cncr.33587

[2] Siegel RL, Giaquinto AN, Jemal A, Cancer statistics. 2024. CA Cancer J Clin. Jan-Feb 2024;74(1):12–49. 10.3322/caac.21820 10.3322/caac.21820 38230766

[3] 2020 Snapshot: State of the oncology workforce in America. JCO Oncol Pract. Jan 2021;17(1):30. 10.1200/op.20.00577. 10.1200/OP.20.00577 32840423

[4] Erikson C, Salsberg E, Forte G, Bruinooge S, Goldstein M. Future supply and demand for oncologists: challenges to assuring access to oncology services. J Oncol Pract Mar. 2007;3(2):79–86. 10.1200/jop.0723601. 10.1200/JOP.0723601 PMC2793740 20859376

[5] U.S. Department of veterans affairs. Rural veterans: 2021–2023. Accessed September 9. 2024.

[6] Yen TWF, Pan IW, Shih YT. Impact of state telehealth policies on telehealth use among patients with newly diagnosed cancer. JNCI Cancer Spectr Aug. 2023;31(5). 10.1093/jncics/pkad072. 10.1093/jncics/pkad072 PMC10597585 37713464

[7] Cascella L. Virtual Risk: an overview of telehealth from a risk management perspective. Disponibile online su www medpro com/documents/10502/2820774/Virtual+ Risk+-+ An+ Overview + of+ Telehealth pdf (ultimo accesso novembre 2015). 2014.

[8] Rutledge CM, Kott K, Schweickert PA, Poston R, Fowler C, Haney TS. Telehealth and eHealth in nurse practitioner training: current perspectives. Adv Med Educ Pract. 2017;8:399–409. 10.2147/amep.S116071. 28721113 10.2147/AMEP.S116071 PMC5498674

[9] Heudel PE, Ait Ichou M, Favier B, Crochet H, Blay JY. Can Digital Health Improve Therapeutic Compliance in Oncology? JCO Clin Cancer Inf Oct. 2024;8:e2400205. 10.1200/cci-24-00205. 10.1200/CCI-24-00205 39454112

[10] Kruse CS, Pacheco GJ, Vargas B, Lozano N, Castro S, Gattu M. Leveraging telehealth for the management of breast cancer: A systematic review. Healthc (Basel) Oct. 2022;12(10). 10.3390/healthcare10102015. 10.3390/healthcare10102015 PMC9602569 36292461

[11] Vanderpool RC, Muro A, Jensen RE. Advancing a telehealth and cancer control research agenda at the US National Cancer Institute. J Natl Cancer Inst Monogr Jun. 2024;26(64):51–4. 10.1093/jncimonographs/lgae016. 10.1093/jncimonographs/lgae016 PMC12104160 38924788

[12] Annunziata CM, Dahut WL, Willman CL, Winn RA, Knudsen KE. Reflections on the state of telehealth and cancer care research and future directions. J Natl Cancer Inst Monogr Jun. 2024;26(64):100–3. 10.1093/jncimonographs/lgae008. 10.1093/jncimonographs/lgae008 38924793

[13] Jensen RE, Brick R, Medel J, et al. National Cancer Institute-funded grants focused on synchronous telehealth cancer care delivery: a portfolio analysis. J Natl Cancer Inst Monogr Jun. 2024;26(64):55–61. 10.1093/jncimonographs/lgae003. 10.1093/jncimonographs/lgae003 PMC11207831 38924791

[14] Zullig LL, Raska W, McWhirter G, et al. Veterans Health Administration National TeleOncology Service. JCO Oncol Pract Apr. 2023;19(4):e504–10. 10.1200/op.22.00455. 10.1200/OP.22.00455 PMC10113113 36649579

[15] Lewinski AA, Crowley MJ, Miller C, et al. Applied Rapid Qualitative Analysis to Develop a Contextually Appropriate Intervention and Increase the Likelihood of Uptake. Med Care Jun. 2021;1(Suppl 3):S242–51. 10.1097/mlr.0000000000001553. 10.1097/MLR.0000000000001553 PMC8132894 33976073

[16] Damschroder LJ, Aron DC, Keith RE, Kirsh SR, Alexander JA, Lowery JC. Fostering implementation of health services research findings into practice: a consolidated framework for advancing implementation science. Implement Sci Aug. 2009;7:4:50. 10.1186/1748-5908-4-50. 10.1186/1748-5908-4-50 PMC2736161 19664226

[17] Abraham TH, Finley EP, Drummond KL, et al. A method for developing trustworthiness and preserving richness of qualitative data during team-based analysis of large data sets. Am J Evaluation. 2021;42(1):139–56.

[18] Sandelowski M. Whatever happened to qualitative description? Res Nurs Health Aug. 2000;23(4):334–40. 10.1002/1098-240x(200008)23:4<334::aid-nur9>3.0.co;2-g 10940958

[19] Sandelowski M. What’s in a name? Qualitative description revisited. Res Nurs Health Feb. 2010;33(1):77–84. 10.1002/nur.20362. 10.1002/nur.20362 20014004

[20] Averill JB. Matrix analysis as a complementary analytic strategy in qualitative inquiry. Qual Health Res Jul. 2002;12(6):855–66. 10.1177/104973230201200611. 10.1177/104973230201200611 12109729

[21] Elliott V. Department of veterans affairs (VA): a primer on telehealth. Congressional Res Service Rep. 2019.

[22] Chiba A, McWhirter G, Moss H, et al. Implementation of a virtual tumor board through VA National teleoncology service. American society of clinical oncology; 2024.

[23] Jiang CY, El-Kouri NT, Elliot D, et al. Telehealth for Cancer Care in Veterans: Opportunities and Challenges Revealed by COVID. JCO Oncol Pract Jan. 2021;17(1):22–9. 10.1200/op.20.00520. 10.1200/OP.20.00520 32970512

[24] Parikh DA, Rodgers TD, Passero VA, et al. Teleoncology in the Veterans Health Administration: Models of Care and the Veteran Experience. Am Soc Clin Oncol Educ Book Jun. 2024;44(3):e100042. 10.1200/edbk_100042. 10.1200/EDBK_100042 38870449

[25] Zullig LL, Blue C, Kelley MJ, et al. The veterans affairs health care system national teleoncology service: veteran and provider perspectives and experiences. Cancer Survivorship Res Care. 2024;2(1):2329363.

[26] Affairs DoV. VA expands telehealth by allowing health care providers to treat patients across state lines [Press release]. 2018.

[27] Challinor JM, Alqudimat MR, Teixeira TO, Oldenmenger WH. Oncology nursing workforce: challenges, solutions, and future strategies. Lancet Oncol. 2020;21(12):e564–74. 33212044 10.1016/S1470-2045(20)30605-7

[28] Shulman LN, Sheldon LK, Benz EJ. The future of cancer care in the United States—overcoming workforce capacity limitations. JAMA Oncol. 2020;6(3):327–8. 31971546 10.1001/jamaoncol.2019.5358

